# p38 mitogen-activated protein kinase determines the susceptibility to cigarette smoke-induced emphysema in mice

**DOI:** 10.1186/1471-2466-14-79

**Published:** 2014-05-07

**Authors:** Satoshi Marumo, Yuma Hoshino, Hirofumi Kiyokawa, Naoya Tanabe, Atsuyasu Sato, Emiko Ogawa, Shigeo Muro, Toyohiro Hirai, Michiaki Mishima

**Affiliations:** 1 Department of Respiratory Medicine, Graduate School of Medicine, Kyoto University, 54 Kawahara-cho, Shogoin, Sakyo-ku, Kyoto, Japan

**Keywords:** Chronic obstructive pulmonary disease, Animal model, Disease susceptibility, Signal transduction, Molecular targeted therapy

## Abstract

**Background:**

There is a need for agents that suppress inflammation and progression of chronic obstructive pulmonary disease. p38 mitogen-activated protein kinase (p38 MAPK) has been associated with this disorder, and several inhibitors of this cascade are in clinical trials for its treatment, but their efficacy and utility are unknown. This study evaluated the relationship between p38 MAPK activation and susceptibility to cigarette smoke (CS)-induced emphysema, and whether its inhibition ameliorated the lung inflammation and injury in murine models of cigarette smoke exposure.

**Methods:**

In acute and chronic CS exposure, the activation and expression of p38 MAPK in the lungs, as well as lung inflammation and injury (proteinase production, apoptosis, and oxidative DNA damage), were compared between two mouse strains: C57BL/6 (emphysema-susceptible) and NZW (emphysema-resistant). The selective p38 MAPK inhibitor SB203580 (45 mg/kg) was administrated intra-peritoneally to C57BL/6 mice, to examine whether it ameliorated cigarette smoke-induced lung inflammation and injury.

**Results:**

Acute CS-induced lung inflammation (neutrophil infiltration, mRNA expressions of TNF-α and MIP-2), proteinase expression (MMP-12 mRNA), apoptosis, and oxidative DNA damage were significantly lower in NZW than C57BL/6 mice. p38 MAPK was significantly activated and up-regulated by both acute and chronic CS exposure in C57BL/6 but not NZW mice. mRNA expression of p38 MAPK was also upregulated in C57BL/6 by chronic CS exposure and tended to be constitutively suppressed in NZW mice. SB203580 significantly attenuated lung inflammation (neutrophil infiltration, mRNA expressions of TNF-α and MIP-2, protein levels of KC, MIP-1α, IL-1β, and IL-6), proteinase expression (MMP-12 mRNA), oxidative DNA damage, and apoptosis caused by acute CS exposure.

**Conclusions:**

Cigarette smoke activated p38 MAPK only in mice that were susceptible to cigarette smoke-induced emphysema. Its selective inhibition ameliorated lung inflammation and injury in a murine model of cigarette smoke exposure. p38 MAPK pathways are a possible molecular target for the treatment of chronic obstructive pulmonary disease.

## Background

Chronic obstructive pulmonary disease (COPD) is the fourth leading cause of death worldwide [1], and further increases in its prevalence and mortality are predicted [2]. COPD is characterized by airway obstruction and progressive lung inflammation associated with the influx of inflammatory cells [3]. The inflammation in the respiratory tract appears to be an amplification of the normal response to chronic irritants such as cigarette smoke (CS). The underlying mechanisms are not understood, but might be genetically determined. Lung inflammation is further amplified by oxidative stress and excess proteinases in the lung. These mechanisms lead to characteristic COPD pathological changes [4]. Although emphysema can be developed without enhancing inflammation in some animal models [5,6], the central pathogenesis of human COPD is still believed to be chronic lung inflammation.

There is limited evidence that regular treatment with long-acting β2-agonists, inhaled corticosteroids, and combinations of these will decrease the rate of decline of lung function [7]. However, most studies have indicated that existing medications for COPD do not modify the long-term decline in lung function that is the hallmark of this disease [8-11], and only decrease symptoms and/or complications. Corticosteroids have widely been used in an attempt to modulate the chronic inflammatory response and eventually stop disease progression. However, they are largely ineffective in attenuating inflammation in COPD patients [12]. Corticosteroid resistance might involve the impaired activity of the enzyme histone deacetylase, and is probably related to oxidative stress [13]. Several alternative anti-inflammatory approaches, such as anti-tumor necrosis factor (TNF) and phosphodiesterase (PDE)-4 inhibitors, are being investigated for COPD treatment, but have been unsuccessful to date [14,15]. There is a pressing need for more effective anti-inflammatory drugs for the treatment of COPD.

Inflammatory signals are generally initiated by the activation of multiple cell-surface receptors, then a limited number of kinase-signaling molecules, followed by numerous effector molecules [16-18]. Novel therapeutics might target the most common molecules associated with COPD, such as kinases. Indeed, activation of p38 mitogen-activated protein kinase (MAPK) has been associated with COPD in humans [19]. A p38 MAPK inhibitor was also shown to inhibit CS-induced inflammation in a murine model [20]. It remains unclear whether such anti-inflammatory effects are sufficient for suppressing the pathogenesis responsible for CS-induced lung inflammation, and subsequent emphysema development [21-23].

Here we used a murine model of CS exposure to evaluate the significance of p38 MAPK activation in COPD pathogenesis and its potential as a molecular target for therapeutics. We compared MAPK activation by CS exposure between two murine strains with different susceptibility to emphysema. We then explored the effects of the specific p38 MAPK inhibitor SB203580 on CS-induced oxidative DNA damage, apoptosis, excessive protease production, and lung inflammation.

## Methods

### Animals

Male C57BL/6 (emphysema-susceptible) and NZW (emphysema-resistant) mice (6–8 weeks old) were purchased from Japan SLC (Shizuoka, Japan). The mice were housed in a temperature-controlled conventional room, and supplied with laboratory chow and water *ad libitum* for at least 4 weeks before starting the smoke exposure. The study protocol was approved by the Animal Research Committee of Kyoto University, Japan.

### CS exposure

**Figure 1:**
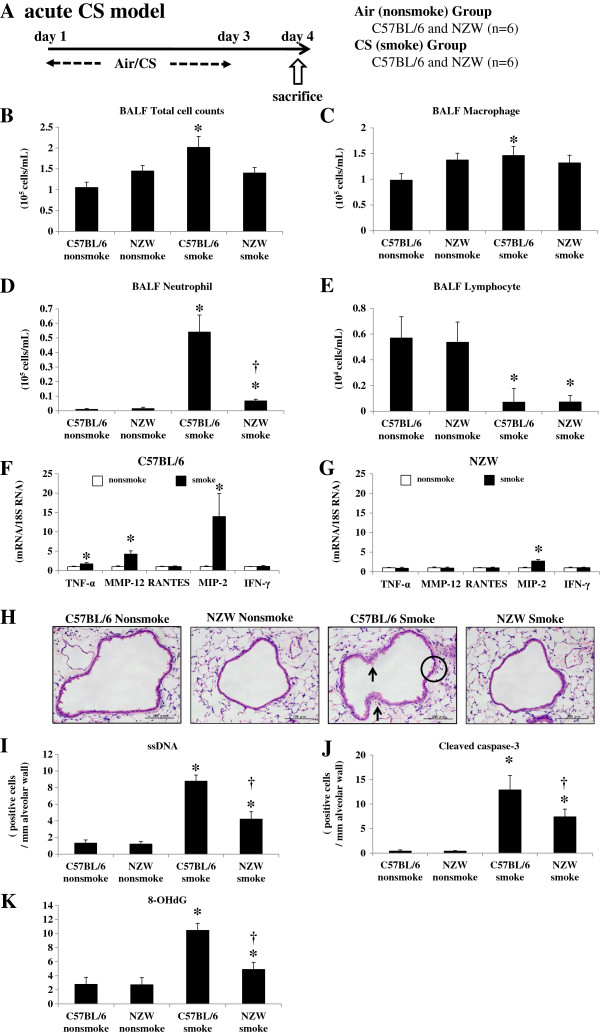
**Acute cigarette smoke model. A**. To investigate the relationship between p38 MAPK activation and lung inflammation and injury after CS exposure, C57BL/6 and NZW mice were exposed to air (no-smoke group) or CS for 3 days (n = 6). **B**-**E**. Inflammatory cell counts in BALF. BALF total cell **(B)**, macrophage (C) and neutrophil counts **(D)** were significantly increased by CS exposure in C57BL/6 mice, but to a lesser degree or not at all in NZW mice. BALF lymphocyte counts were significantly decreased by CS exposure in both strains **(E)**. **F**.**G**. mRNA expression of inflammatory mediators in the lungs. The expression of 18S rRNA was used as an internal control. mRNA expression levels of TNF-α, MIP-2, and MMP-12 were significantly up-regulated by CS exposure in C57BL/6 mice **(F)**, but to a lesser degree or not at all in NZW mice **(G)**. **H**. Histological lung differences after CS exposure between C57BL/6 and NZW mice. Mouse lungs exposed to CS demonstrated cell death, seen as cytoplasmic vacuolization (circle) and cytoplasmic blebbing (arrow) of the bronchial epithelium. Acute CS exposure induced these changes in C57BL/6 mice but to a lesser degree in NZW mice. **I**. **J**. Apoptosis in the lungs following CS exposure assessed by immunohistochemistry. There were significantly fewer apoptotic cells in NZW mice, as represented by ssDNA **(I)** and cleaved caspase-3 **(J)**-positive cells, compared with C57BL/6 mice. **K**. Oxidative stress following CS exposure evaluated by increased 8-OHdG levels of lung DNA using an ELISA. CS exposure caused a marked increase in 8-OHdG levels of mouse lungs in both strains, but to a lesser extent in NZW than in C57BL/6 mice. *p < 0.05 compared with corresponding non-smoke groups. †*p* < 0.05 compared with C57BL/6 smoke groups. n = 6 for each experimental set.

**Figure 2:**
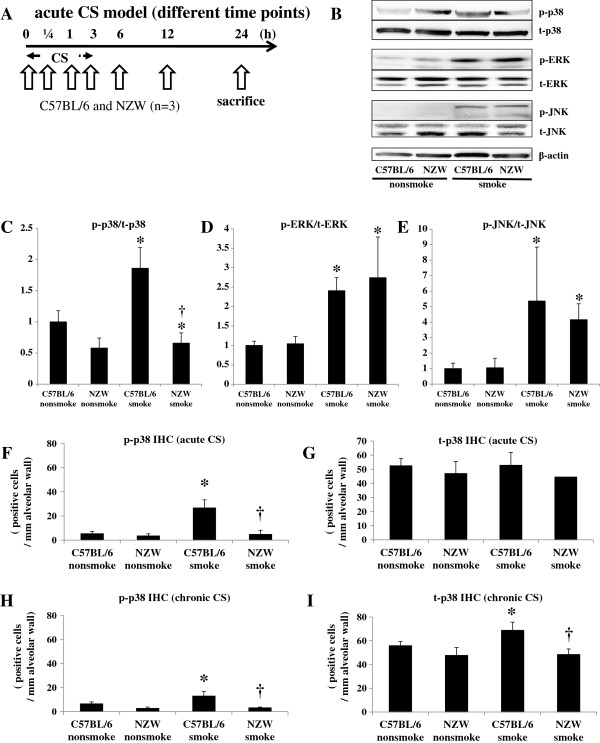
**p38 MAPK activation. A**. To assess MAPK activation, C57BL/6 and NZW mice were exposed to acute CS, and sacrificed at 0 h, 0.25 h, 1 h, 3 h, 6 h, 12 h, and 24 h from the start of CS exposure. **B**-**E**. Phosphorylated and total levels of p38 MAPK, ERK, and JNK in the lungs were analyzed by western blotting, with β-actin as an indicator for equal protein loading. Phosphorylation of p38 MAPK in the lungs was confirmed in C57BL/6 mice, but not in NZW mice. Phosphorylation of ERK and SAPK/JNK was noted in both strains in response to CS exposure. Western blots are representative of three independent experiments evaluating murine lungs at 1 hr after the start of acute CS exposure **(C, D, E)**. The intensities of the electrophoretic bands were quantified and expressed as p-MAPK/t-MAPK. p-MAPK, phosphorylated-MAPK; t-MAPK, total MAPK. *p < 0.05 compared with corresponding non-smoke groups. †*p* < 0.05 compared with C57BL/6 smoke groups. n = 3 for each experimental set. **F**-**I**. Phosphorylated and total p38 MAPK following acute CS exposure were evaluated by immunohistochemistry. Acute CS exposure caused a marked increase in the number of phosphorylated p38-positive cells in the alveolar walls of C57BL/6 mice, but not NZW mice **(F)**. Total numbers of p38-positive cells were not increased by acute CS exposure **(G)**. Chronic CS exposure caused a marked increase in the numbers of both phosphorylated and total p38-positive cells in the alveolar walls of C57BL/6 mice, but not NZW mice **(H, I)**. p-p38, phosphorylated-p38; t-p38, total p38. *p < 0.05 compared with corresponding non-smoke groups. †*p* < 0.05 compared with C57BL/6 smoke groups. n = 6 for each experimental set.

**Figure 3:**
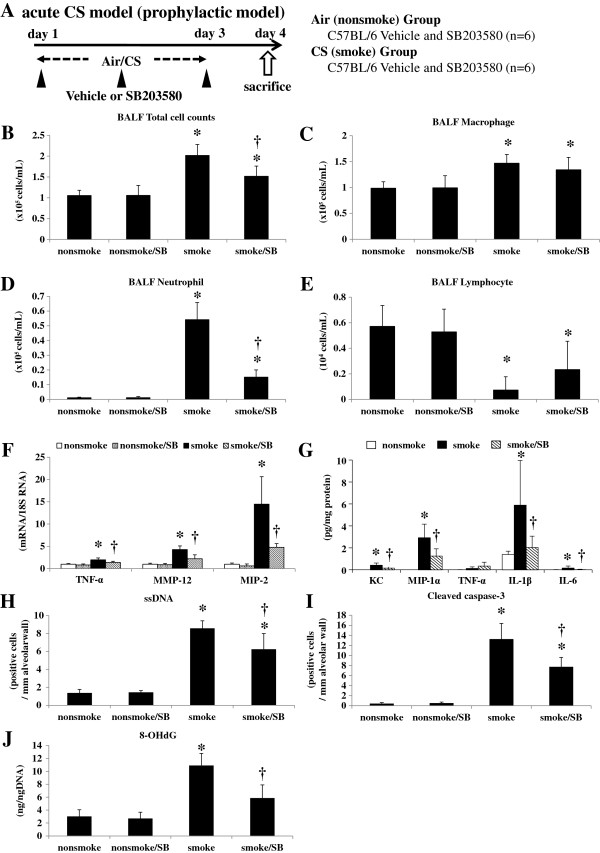
**Acute cigarette smoke model (prophylactic model). A**. To explore the effects of a specific p38 MAPK inhibitor, C57BL/6 mice were exposed to air or CS for 3 days, and were treated by intra-peritoneal injection with vehicle (dimethysulfoxide) or SB203580 (45 mg/kg) 30 min before every CS exposure for 3 days as prophylaxis (n = 6). **B**-**E**. SB203580 significantly suppressed the increase in total cell counts and neutrophil counts in BALF. **F**. SB203580 significantly suppressed the lung mRNA expression levels of TNF-α, MMP-12, and MIP-2. **G**. SB203580 significantly suppressed the lung protein levels of KC, MIP-1α, IL-1β, and IL-6. **H**.**I**. SB203580 significantly suppressed the ssDNA-positive and cleaved caspase-3-positive cells in the alveolar septa as assessed by immunohistochemistry. **J**. SB203580 significantly suppressed the lung 8-OHdG production as assessed by an ELISA. *p < 0.05 compared with non-smoke group. †*p* < 0.05 compared with smoke group. n = 6 for each experimental set.

**Figure 4:**
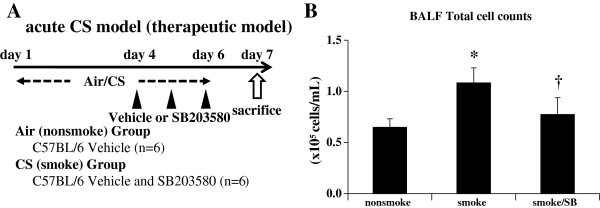
**Acute cigarette smoke model (therapeutic model). A**. As a therapeutic experiment, C57BL/6 mice were exposed to air or CS for 3 days to fully develop lung inflammation and were subsequently treated by intra-peritoneal injection with vehicle (dimethylsulfoxide) or SB203580 (45 mg/kg) 30 min before CS exposure at days 4 to 6. **B**. Therapeutic administration of SB203580 reduced inflammatory cells in BALF. *p < 0.05 compared with non-smoke group. †*p* < 0.05 compared with smoke group. n = 6 for each experimental set.

**Figure 5:**
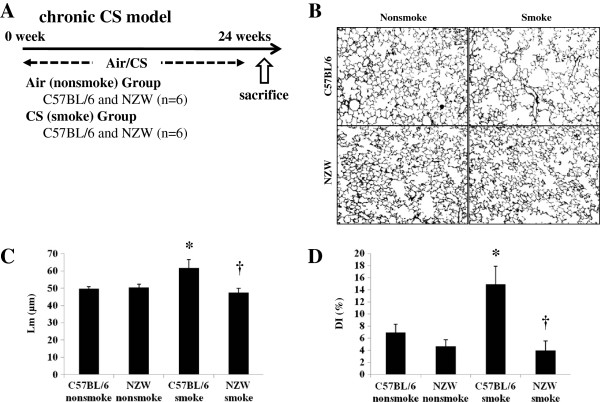
**Chronic cigarette smoke model. A**. C57BL/6 and NZW mice were exposed to air (no-smoke group) or for 24 weeks in the chronic study (n = 6). **B**. Black and white conversion of lung photomicrographs of non-smoke and smoke group mice at 24 weeks. **C-D**. Air-space dilatation and destruction were evaluated by Lm and DI respectively. Both were significantly increased following CS exposure in C57BL/6 but not NZW mice. *p < 0.05 compared with corresponding non-smoke groups. †p < 0.05 compared with C57BL/6 smoke groups. n = 6 for each experimental set.

According to our previous protocol [24], mice were exposed to CS in acute and chronic studies. In both studies, CS was generated by burning filter-cut standard cigarettes (Kentucky 2R4F reference cigarette, Cigarette Laboratory at the Tobacco and Health Research Institute, University of Kentucky, Lexington, KY) using a smoke generator (SG-200, Shibata Scientific Technology Ltd., Tokyo, Japan). CS was diluted to 3% with air to reduce toxicity. In the acute study, mice were exposed to mainstream CS in a Plexiglas box for 1 h daily for 3 or 6 days (40 cigarettes/day) (Figures 1A, 2A, 3A and 4A). In the chronic study, mice were exposed to CS from 10 cigarettes/day, 5 days a week for 24 weeks using a nose-breathing apparatus (Figure 5A). Experiments were performed safely, and no mice were killed through smoke exposure. Blood carboxyhemoglobin (COHb) levels were approximately 30% in the acute study and approximately 15% in the chronic study immediately after CS exposure. They were reduced to 0–1% after 24 h exposure, and there was no daily accumulation through repeated CS exposure. The levels of total particle matter were 395.8 mg/m3 in the acute study and 445.3 mg/m3 in the chronic study.

At 24 h after the last CS exposure, mice were anesthetized with 70 mg/kg pentobarbital by intra-peritoneal injection, and subjected to bronchoalveolar lavage. They were then killed by exsanguination and the lungs were extracted with tracheal cannulation. The right lungs were snap-frozen in liquid nitrogen. The left lungs were fixed with 10% formalin at a constant pressure of 25 cm H_2_O for histological examinations.

### p38 MAPK inhibitor injection

The selective inhibitor of p38 MAPK SB203580 (45 mg/kg; LC laboratories, Woburn, MA) was administered to the C57BL/6 mice, to determine whether it would ameliorate CS-induced lung inflammation and injury. Mice were exposed to CS according to the acute study protocol (40 cigarettes/day for 3 days), and were treated by intra-peritoneal injection with SB203580 or vehicle (dimethylsulfoxide) 30 min before every CS exposure (Figure 2A). A separate experiment was performed to examine the therapeutic effect of SB203580 where mice were exposed to CS for 6 days and treated with SB203580 on days 4 to 6 (Figure 3A).

### Bronchoalveolar lavage (BAL) and the cell differential

Lungs were lavaged five times with 1 ml cold saline through an intratracheal cannula. The lavage fluid was collected and centrifuged to determine the inflammatory cell differential (Shandon Scientific Ltd, Cheshire, UK). At least 600 cells were counted on each cytospin slide stained with Diff-Quik (Dade Behring, Switzerland) under a light microscope.

### RNA isolation and real-time Polymerase Chain Reaction (PCR)

Total RNA was extracted from right lung tissue using TRIzol (Invitrogen, Carlsbad, CA), according to the manufacturer’s instructions. Single-stranded complementary DNA (cDNA) was synthesized from 1 μg total RNA using the SuperScript III Reverse Transcription Kit (Invitrogen). cDNA was amplified and quantified using the Applied Biosystems 7300 Real-Time PCR System (Applied Biosystems, Foster City, CA) with oligonucleotide PCR primer pairs and fluorogenic probes (TaqMan Gene Expression Assay, Applied Biosystems) for TNF-α, matrix metalloproteinase-12 (MMP-12), chemokine (C–C motif) ligand 5 (RANTES), macrophage-inflammatory protein-2 (MIP-2), interferon-γ (IFN-γ) and p38 MAPK (Applied Biosystems catalogue numbers Mm00443258_m1, Mm00500554_m1, Mm01302428_m1, Mm00436450_m1, Mm00801778_m1, and Mm00442491_m1, respectively). 18 s ribosomal RNA (rRNA; Applied Biosystems catalogue number 4310893E) was used as an endogenous control.

### BioPlex cytokine array

In order to examine anti-inflammatory effects of the MAPK inhibitor at a protein level, lung homogenates of C57 mice (non-smoke, CS-exposed, CS-exposed and SB-injected) were subjected to BioPlex cytokine assay (Bio-Rad Laboratories, Richmond, CA). Twenty-three chemokines and cytokines (IL-1α, IL-1β, IL-2, IL-3, IL-4, IL-5, IL-6, IL-9, IL-10, IL-12(p40), IL-12(p70), IL-13, IL-17, Eotaxin, G-CSF, GM-CSF, IFN-γ, KC, MCP-1, MIP-1α, MIP-1β, RANTES, TNF-α) were measured according to the manufacturer’s instruction. Data were normalized with protein concentration.

### 8-hydroxydeoxyguanosine (8-OHdG) Enzyme-Linked Immunosorbent Assay (ELISA)

Total DNA was extracted from right-lung tissue using a QIAamp DNA Mini Kit (Qiagen, Valencia, CA) according to the manufacturer’s instructions. 8-OHdG levels in the DNA samples were analyzed using an ELISA kit (New 8-OHdG Check; Japan Institute for the Control of Aging, Nikken SEIL, Shizuoka, Japan), according to the manufacturer’s instructions. Briefly, 8-OHdG antibody plus sample DNA were added to a 96-well plate precoated with 8-OHdG and incubated overnight at 4°C. The plate was then incubated with horseradish peroxidase-conjugated secondary antibody for 1 h at room temperature followed by 15 min substrate reaction with 3,3′, 5,5′-tetramethylbenzidine. The reaction was terminated by the addition of phosphoric acid, and absorbance was measured at 450 nm. All assays were performed in duplicate and the average concentration of 8-OHdG, normalized per ng total DNA, was calculated for each sample.

### Western blotting for mitogen-activated protein kinases (MAPKs)

To assess MAPK activation, different sets of mice received a single exposure to the same 3% diluted CS, and were then sacrificed as described above at 0 h, 0.25 h, 1 h, 3 h, 6 h, 12 h, and 24 h after the start of CS exposure. Lysates of lung tissue (50 μg protein) from the right lung was subjected to sodium dodecyl sulfate polyacrylamide gel electrophoresis (SDS-PAGE) followed by western blotting with primary antibodies for phosphorylated and total p38 MAPK, phosphorylated and total extracellular signal-regulated kinase (ERK), and phosphorylated and total stress activated protein kinase (SAPK)/c-Jun N-terminal kinase (JNK) (Cell Signaling, Beverly, CA, respectively). Equal loading of the sample was determined by quantitation of protein as well as by reprobing membranes for β-actin (Imgenex, San Diego, CA) as a housekeeping protein. The blots were visualized using enhanced chemiluminescence fluid (ECL plus, Amersham, Buckinghamshire, UK). The intensities of electrophoretic bands were quantified using Quantity One 1-D analysis software (Bio-Rad, Hercules, CA) and expressed as the ratio to β-actin.

### Immunohistochemistry

Apoptosis was assessed by immunohistochemistry according to our previous reports [24]. Briefly, formalin-fixed lung sections were incubated with a rabbit polyclonal anti-single stranded DNA (ssDNA) primary antibody (1:100 dilution; DakoCytomation California Inc., Carpinteria, CA) and a rabbit polyclonal anti-cleaved caspase-3 primary antibody (1:400 dilution; Cell Signaling, Danvers, MA). Staining was performed using the DAKO EnVision + system (peroxidase/3′-diaminobenzidine [DAB]; DAKO, Kyoto, Japan) and counterstained with 1% methylgreen. Immunoreactive cells were counted in at least five fields, and expressed as the positive cell ratio to the length of the alveolar septa.

Immunohistochemistry of p38 MAPK was performed using a rabbit monoclonal primary antibody against the active form of p38 (phospho-p38) MAPK (dilution 1:100; Cell Signaling, Beverly, CA). Staining and counting were performed using the same methods as the apoptosis evaluation.

### Evaluation of lung pathology and quantification of emphysema

The left lungs were fixed with 10% formalin at a constant pressure of 25 cm H_2_O, cut sagittally in 4-μm sections, and stained with hematoxylin and eosin (HE) for histological analysis. Findings were quantified using a four-point scoring system (0, normal; 3, severe) by two analysts blinded to the groups according to a previous method [25]. At least three sections were used for the analysis of each mouse. Periodic acid-Schiff (PAS) stain was performed to evaluate mucus production of airways. For the evaluation of emphysematous change after chronic CS exposure, we calculated the mean linear intercept (Lm) and the destructive index (DI) according to previous methods [24,26].

### Statistical analysis

Results are expressed as means ± standard deviations (SDs). Statistical analysis was performed using JMP software version 6 (SAS institute Inc., Cary, NC). Groups were compared by two-way analysis of variance (ANOVA) followed by Tukey-Kramer’s *post hoc* test. *P* values < 0.05 were considered significant.

## Results

### Acute CS exposure

Lung inflammation and injury were evaluated 24 h after the last CS exposure (Figure 1A). The bronchoalveolar lavage fluid (BALF) total cell and macrophage counts were significantly increased by CS exposure in C57BL/6, but not NZW, mice (Figure 1B, 1C). The BALF neutrophil counts were significantly increased in both strains, but to a significantly lesser extent in NZW mice compared with C57BL/6 mice (Figure 1D). Lymphocytes were significantly decreased in response to CS in both strains (Figure 1E).

Messenger RNA (mRNA) expression levels of the inflammatory cytokines TNF-α and MIP-2 were significantly up-regulated by CS exposure in C57BL/6 mice (1.8-fold and 14.0-fold, respectively), but to a significantly lesser extent in NZW mice (0.88-fold and 2.7-fold, respectively) (Figure 1F, 1G). There was no significant up-regulation of RANTES or IFN-γ by CS exposure in either strain. MMP-12 was also up-regulated by CS exposure (4.3-fold), but to a significantly lesser extent in NZW mice (0.95-fold) (Figure 1F, 1G).

The histology of C57BL/6 mice exposed to CS revealed severe lung injury in the form of cytoplasmic vacuolization and cytoplasmic blebbing of the bronchial epithelium indicating necrotic cell death (Figure 1H). The NZW mice showed significantly less severe cytoplasmic vacuolization (0.99 ± 0.52 vs. 1.74 ± 0.45, respectively) and blebbing (1.13 ± 0.46 vs. 2.61 ± 0.60, respectively) than C57BL/6 mice, according to a semi-quantitative histological analysis. There was not mucus overproduction evaluated by PAS stain in the acute CS exposure model (Additional file 1: Figure S1C).

The apoptosis of lung cells was also enhanced by CS exposure in both strains of mice, as represented by an increased number of single-stranded DNA (ssDNA)-positive or cleaved caspase-3-positive cells (Additional file 1: Figure S1A, 1B). Apoptotic cells were mainly localized to the alveolar septa. The NZW mice had significantly fewer ssDNA-positive and cleaved caspase-3-positive cells compared with the C57BL/6 mice after CS exposure (Figure 1I, 1 J).

Oxidative DNA damage in the lungs was markedly enhanced in the C57BL/6 mice by CS exposure, as represented by increased 8-OHdG levels in lung DNA (Figure 1K). The oxidative DNA damage levels were significantly lower in the NZW mice after CS exposure.

### Chronic CS exposure

C57BL/6 and NZW mice were exposed to air (no-smoke group) or for 24 weeks in the chronic study (n = 6) (Figure 5A). Air-space dilatation and destruction were evaluated by Lm and DI respectively. Both were significantly increased following CS exposure in C57BL/6 but not NZW mice (Figure 5B, 5C, 5D). There was not mucus overproduction evaluated by PAS stain in the chronic CS exposure model (Additional file 1: Figure S3C).

### p38 MAPK activation

In preliminary acute CS time course experiment (n = 1), the phosphorylation of p38 MAPK in the lungs was confirmed at 0.25 h, 1 h, 3 h, and 6 h after the start of CS exposure in C57BL/6 mice, but was not seen in NZW mice even at 24 h after exposure (Figure 2A, Additional file 1: Figure S2A). Notably, the baseline levels (without CS exposure) of total and phosphorylated p38 MAPK were much lower in NZW mice than C57BL/6 mice. By contrast, the phosphorylation of ERK and SAPK/JNK was noted in both strains of mice in response to CS exposure. Then, we performed three independent experiments evaluating murine lungs at 1 hr after the start of acute CS exposure. Western blots are representative of three independent experiments (Figure 2B). The intensities of the electrophoretic bands were quantified and expressed as p-MAPK/t-MAPK (Figure 2C, 2D, 2E). p38 MAPK activation were not detected in chronic (24 wk) models by Western blots (Additional file 1: Figure S1B).

Immunohistochemical analysis revealed that acute CS exposure (3 d) markedly increased the number of phospho-p38-positive cells in the alveolar walls, and possibly the macrophages and pneumocytes, in C57BL/6 mice, but not in NZW mice (Figure 2F, Figure 2G, Additional file 1: Figure S2C). In the chronic study, the number of phospho-p38-positive cells was also significantly increased in C57CL/6 mice (198% of control), but not in NZW mice (113% of control) in the chronic study (Figure 2H, 2I).

The mRNA levels of p38 MAPK were significantly up-regulated by CS exposure in C57BL/6 mice in the chronic study, but not in the acute study (Additional file 1: Figure S2E, S2I). There was also no significant up-regulation of p38 MAPK mRNA expression levels in NZW mice, but they were significantly lower than those in C57BL/6 mice after chronic CS exposure. The expression levels of MMK3, MMK6 and MAPKAPK-2 were not up-regulated in acute CS exposure (Additional file 1: Figure S2F-H).

### Acute CS model (prophylactic and therapeutic model)

Administration of the selective p38 MAPK inhibitor SB203580 significantly suppressed the increase in total cell counts and BALF neutrophils following 3 days of CS exposure (by 52.1% and 73.6%, respectively) (Figure 3B, 3D). Lung injury due to acute CS exposure was ameliorated by injected SB203580: there was significantly less cytoplasmic vacuolization (1.31 ± 0.21 vs. 1.82 ± 0.48, respectively) and blebbing (1.76 ± 0.55 vs. 2.70 ± 0.84, respectively) in mice injected with SB203580 compared with controls, as evaluated by the histological lung injury score. SB203580 significantly reduced the up-regulation of TNF-α, MIP-2, and MMP-12 mRNA expression levels (by 60.1%, 62.6%, and 71.9%, respectively) (Figure 3F). Protein levels of chemokines and pro-inflammatory cytokines such as KC, MIP-1α, IL-1β, and IL-6 were elevated in the lungs of C57BL/6 mice in response to CS exposure and SB203580 significantly suppressed the augmentation (by 36.7%, 42.8%, 14.1%, and 11.7%, respectively) (Figure 3G). The other 19 cytokines examined including TNF-α were not affected by CS exposure. SB203580 also significantly reduced the increase in ssDNA-positive or cleaved caspase-3-positive apoptotic cells (by 32.3% and 43.0%, respectively) (Figure 3H, 3I). 8-OHdG production induced by acute CS exposure was significantly attenuated by the administration of SB203580 (by 64.0%) (Figure 3J).

In addition to prophylaxis, therapeutic effects of SB203580 were examined where SB203580 successfully attenuated BALF inflammatory cells by 28.8% (Figure 4B).

## Discussion

This study demonstrated that cigarette smoking activated p38 MAPK only in mice that were susceptible to CS-induced emphysema, and that the selective inhibition of p38 MAPK ameliorated lung injury and inflammation in a murine model of CS exposure. Lung inflammation, proteinase production, apoptosis, and oxidative stress were markedly activated in susceptible C57BL/6 mice, but less so in resistant NZW mice, and this was paralleled by the activation of p38 MAPK in both the acute and chronic studies. These results suggest a relationship between p38 MAPK activation and susceptibility to CS-induced emphysema. Moreover, the selective p38 MAPK inhibitor SB203580 significantly ameliorated lung inflammation, proteinase production, apoptosis, and oxidative DNA damage in C57BL/6 mice. These results might establish the basis for using p38 MAPK pathways as novel molecular targets for the treatment of COPD.

The present study evaluated the significance of p38 MAPK activation in COPD pathogenesis and its potential as a molecular target in COPD therapeutics. In recent years, steps have been taken to delineate the intracellular signaling cascades that mediate inflammation, in order to clarify the pathogenesis of various inflammatory diseases and to develop novel therapeutics. Much attention has been given to members of the MAPK superfamily due to their consistent activation by pro-inflammatory cytokines, and their role in nuclear signaling. This superfamily includes ERKs (also known as p42/p44), JNKs (also known as SAPKs) and p38 MAPK (also known as cytokine-suppressive binding protein or CSBP). ERKs are activated by growth factors and mitogenic stimuli, whereas p38 and JNK are regulated by stress-inducing signals (such as ultraviolet irradiation and osmotic shock) and pro-inflammatory cytokines [27]. Interest in the p38 family has been particularly intense following the discovery that p38 MAPK inhibitors have an anti-inflammatory effect in models of arthritis and inflammatory angiogenesis *in vivo*, suppressing the expression of inflammatory cytokines, including interlekin-8 (IL-8), TNF-α, and MMPs [28-30].

An association between COPD and the MAPK pathway was suggested by Yao *et al*., who reported that both phosphorylated and total levels of p38 MAPK increased in the lungs of C57BL/6 mice in response to acute CS exposure [31]. Activation of this pathway was also detected in human COPD by Renda *et al.*[19]; they observed that active phosphorylated p38-positive alveolar macrophages and alveolar wall cells were increased in patients with severe and mild/moderate COPD, compared with smoking and nonsmoking controls. Although these studies suggest an association of p38 MAPK activation and COPD, the causal relationship between the two remains unclear. One approach to understanding this is to use an animal model to identify differences in smoke-induced changes between individuals who do or do not go on to develop emphysema. We therefore compared emphysema-susceptible C57BL/6 and resistant NZW mouse strains by subjecting them to short-term CS exposure.

Major COPD pathogenesis, including lung inflammation, apoptosis, oxidative DNA damage, and proteinase expression, was enhanced only in the susceptible strain after 3 days of CS exposure (Figure 1). In addition, 24 weeks of CS exposure caused emphysema only in the same susceptible strain (Figure 5). These results suggest that our animal model was suitable for emulating COPD. p38 MAPK activation varied greatly between the two strains soon after CS exposure, indicating that the inter-strain difference was not a consequence, but rather a cause, of the disease (Additional file 1: Figure S2A). This was corroborated by the experiments using a p38 MAPK inhibitor (Figures 3, 4). However, similar inter-strain differences were not observed for ERK or JNK, suggesting that the up-regulation of these cascades by CS exposure might be independent of emphysema development. We therefore speculate that p38 MAPK is critical for the initiation of the cascade of events leading to emphysema.

In the present study, the phosphorylation of p38 MAPK of the whole lung was detected at one hour from the beginning of CS exposure, but it was not detected after three days CS exposure in acute CS model, whereas the phosphorylation in IHC was detected after three days CS exposure in acute CS model. The discrepancy of the phosphorylation of p38 MAPK between WB and IHC was probably due to the cell source. Our IHC analysis revealed that p38 MAPK was activated in alveolar wall cells. Therefore, p38 MAPK activation was diluted in the whole lung analysis such as WB, resulting in that p38 MAPK activation in WB was detected only in very short time course with intense lung inflammation. CS-induced p38 MAPK was also regulated at the mRNA level. Significant differences were found in the expression of p38 MAPK mRNA between the two strains after CS exposure after the development of emphysema (Additional file 1: Figure S2I). Baseline p38 MAPK mRNA expression level evaluated by realtime PCR is higher in C57BL/6 than NZW, which may reflect higher total p38 MAPK level evaluated by IHC in C57BL/6 than in NZW. Acute CS exposure induced short time intense inflammation with significant phosphorylation of p38 MAPK in C57BL/6, but without up-regulation of p38 MAPK mRNA. Chronic CS exposure induced long term mild inflammation with up-regulation of p38 MAPK mRNA in C57BL/6. MAPKs are generally activated by the phosphorylation of threonine and tyrosine residues within a signature sequence T-X-Y (single letter code) by a dual-specificity MAPK kinase (MEK or MKK) [32]. Therefore, this activation can be evaluated as phosphorylated MAPK/total MAPK. Although transcriptional regulation of p38 MAPK has not been reported, similar regulation of the ERK signaling pathway (MEK2) was previously observed [33]. Clarification of p38 MAPK transcriptional regulation would allow an alternative approach to COPD therapeutics to be developed.

The differences in p38 MAPK expression between susceptible and resistant strains suggest that p38 MAPK expression might be useful as a biomarker of COPD, and more specifically as a disease predictor. This is because the differences were observed regardless of smoke exposure and before the development of emphysema. The detection of p38 MAPK activation in humans could be carried out non-invasively using material such as induced sputum or peripheral whole blood, and could be useful for predicting disease susceptibility. This potential is currently under investigation in our department. Moreover, once p38 MAPK inhibitors are established as COPD therapeutics, the monitoring of p38 MAPK activity could also predict therapeutic responses and disease management.

As MAPKs are involved in critical steps for many inflammatory signals, they are promising therapeutics for a wide variety of inflammatory diseases. Medicherla *et al.* reported the first anti-inflammatory effect of a p38 MAPK inhibitor in a murine model of CS exposure [20]. They found that the selective p38α inhibitor SD-282 successfully ameliorated CS-induced lung inflammation measured by the cell differential using bronchoalveolar lavage, lung histology, and the pro-inflammatory cytokines cyclooxygenase-2 (COX-2) and IL-6. However, it is not clear whether the anti-inflammatory effect of p38 MAPK inhibitors is sufficient to prevent emphysema development. In murine models of CS-induced emphysema, it can take as long as 24 weeks to develop emphysema [34], and it is difficult to inhibit p38 MAPK for such a prolonged period. The aim, therefore, is to identify surrogate markers for therapeutic responses in acute studies that directly suggest protection against lung destruction. Smoke-induced changes such as lung cell apoptosis, oxidative DNA damage, and proteinase expression would be ideal surrogate markers because they were shown in the present study to be up-regulated by short-term smoke exposure only in the susceptible mouse strain, and are already associated with the pathogenesis of human COPD [21-23]. Systemic administration of SB203580 in the present study significantly ameliorated not only CS-induced inflammation as represented by BALF neutrophils, lung mRNA of TNF-α and MIP2, and lung protein of KC, MIP-1α, IL-1β and IL-6 but also proteinase expression as measured by lung MMP-12, apoptosis of alveolar septal cells as demonstrated by ssDNA, and cleaved caspase-3 immunostaining and oxidative DNA damage as measured by 8-OHdG (Figure 3J). Discrepancy between mRNA and protein expressions of TNF-α in response to acute CS was observed in the present study. This discrepancy was also noted in our previous study [24] and it is speculated that cleaved form of TNF-α, but not total content of TNF-α in the lung, might be important for triggering inflammation. Moreover, therapeutic administration of the MAPK inhibitor is sufficient to inhibit lung inflammation caused by acute CS exposure (Figure 4B). Taken together, these results might provide a further basis for p38 MAPK inhibition in COPD therapeutics.

It is not clear how the p38 MAPK inhibitor suppressed smoke-induced changes leading to lung destruction. However, recent studies revealed that the p38 MAPK pathway regulates apoptosis, inflammation, and fibrosis, which are potentially associated with COPD pathogenesis [16,35,36]; 1) inflammatory neutrophil cell migration, 2) proinflammatory cytokine and chemokine release from inflammatory cells and airway smooth muscle, 3) release of degradative enzymes (eg, MMPs) and growth factors, 4) control of the production of interferon-γ from CD4 positive andCD8 positive T cells, and T-helper 1 differentiation of CD4 positive cells, 5) enhancement of bronchoconstrictor effects of airway smooth muscle associated with inflammation and oxidative stress, 6) airway remodeling, 7) induction of corticosteroid insensitivity. Moreover, inhaled CS stimulates epithelial cells and alveolar macrophages to release several chemotactic factors that attract inflammatory cells to the lungs, including neutrophils, T-helper 1 cells, type 1 cytotoxic T cells, and fibroblasts. These inflammatory cells, together with macrophages and epithelial cells, release proteases, growth factors, and pro-inflammatory cytokines, causing chronic lung inflammation and structural changes [37]. This inflammation causes *secondary* oxidative stress. In the present study, immunohistochemical data indicated that CS activated the p38 MAPK signaling pathway in the alveolar wall cells and bronchial epithelial cells of C57BL/6 mice. Therefore, the administration of SB203580 might ameliorate apoptosis and proteinase production via this pathway in these cells. Further investigation is needed to clarify the mechanism.

p38 MAPK activation and oxidative DNA damage were significantly greater in CS susceptible strain than in CS resistant strain in the present study. Moreover, p38 MAPK inhibition ameliorated CS-induced oxidative DNA damage in the lung, suggesting that p38 MAPK activation induces oxidative DNA damage in the CS exposure model. On the other hand, previous papers shown that oxidative stress induced by CS activates p38 MAPK signaling pathways of the lung [38,39]. We might explain the complex mechanisms of cigarette smoke-induced inflammation as follows; CS-induced oxidative stress itself primarily activates p38 MAPK in lung cells, followed by promoting neutrophils recruitment and secondary oxidative stress. Further investigation is needed to clarify the mechanism.

p38 MAPK is reported to regulate mucus overproduction. Although PAS positive cells were detected in the lungs of C57BL/6 mice after 8wk smoke-exposure in previous publication [31], unfortunately, PAS positive cells were not found in our C57BL/6 mice after 6 months smoke exposure. Possible reasons are that 1) we used different substrains for the experiments (we used C57BL/6NCrSlc supplied from Japan SLC), 2) 6 months smoke caused squamous formation in airway epithelial cells (data not shown).

Several p38 MAPK inhibitors have been entered in clinical trials for chronic inflammatory diseases such as rheumatoid arthritis, and inflammatory bowel diseases [40,41]. MacNee *et al.* reported a clinical trial in COPD patients of the orally administrated p38 MAPK inhibitor PH-797804 showing significant improvement of lung function and respiratory symptoms [42]. Notably, medium dose demonstrated the highest effects in the evaluation of the dose–response effects. Singh explained that the bell-shaped dose response curve might be due to another MAPK (ERK or JNK) pathway activation by strongly blocking p38 MAPK pathway [43]. Therefore, optimal dose setting is important for p38 MAPK inhibitors. p38 MAPK inhibitors have encountered major problems in terms of side effects and toxicity, indicating that it might be necessary to administer these drugs by topical application such as inhalation to reduce systemic exposure, or to target downstream substrates such as MAPK-activated protein kinase-2 (MAPKAPK-2). MAPKAPK-2 was reported to be essential for lipopolysaccharide (LPS)-induced endotoxic shock [44]. Although p38 MAPK-knockout mice are embryonic lethal, MAPKAPK-2-knockout mice have a normal lifespan, indicating the safety of inhibiting this substrate. Alternatively, suppression of p38 MAPK at a transcriptional level, as observed in NZW mice, might be a safe approach. NZW mice appear to avoid unnecessary inflammation by maintaining total p38 at lower levels, thus ensuring a minimal defense response. Indeed, no reports have suggested that NZW mice are susceptible to infection.

The present study had some limitations. First, p38 MAPK activation/inhibition was examined in only one susceptible strain, although it was compared with a resistant strain. The roles of p38 MAPK are reported to be different not only between strains but also between cell types and stimulation. As suggested by humans and animal models [45], the pathogenesis of COPD/emphysema is heterogeneous, so it would be preferable to examine the effect of p38 MAPK inhibition in multiple susceptible strains. However, the fact that lung p38 MAPK is present at higher levels in COPD patients than in healthy subjects suggests that p38 activation is a common feature in COPD. p38 inhibition might therefore be successful in patients with higher levels of p38 MAPK activation. Second, the effect of p38 inhibition was examined only in acute CS exposure. There remains a need to explore whether CS-induced emphysematous changes could be ameliorated by the administration of p38 MAPK inhibitors. Our study showed, however, that SB203580 could ameliorate not only lung inflammation but also excessive proteinase production, oxidative DNA damage, and apoptosis, indicating the further possibility of using p38 MAPK inhibitors as a new drug for the treatment of COPD. Alternatively, a chronic smoke study using mice genetically modified in the p38 MAPK pathway might provide additional information. Third, we investigated only whether p38 MAPK inhibition could ameliorate the “CS-induced” development of COPD. It remains unclear whether p38 MAPK inhibition can suppress the progression of COPD that persists after smoking cessation. Although airway inflammation continues after cessation and emphysema still progresses [46], further investigation is needed to understand this.

## Conclusions

CS activated p38 MAPK only in a mouse strain that was susceptible to CS-induced emphysema, and its selective inhibition ameliorated lung inflammation and injury in a murine model of CS exposure. These results demonstrate the significance of p38 MAPK activation in COPD pathogenesis, and might establish a basis for using MAPK pathways as a new molecular target for the treatment of COPD.

## Abbreviations

8-OHdG: 8-hydroxydeoxyguanosine; BALF: Bronchoalveolar lavage fluid; COPD: Chronic obstructive pulmonary disease; CS: Cigarette smoke; DI: Destructive index; ERK: Extracellular signal-regulated kinase; JNK: c-Jun N-terminal kinase; Lm: Mean linear intercept; p38 MAPK: p38 mitogen-activated protein kinase; SAPK: Stress activated protein kinase.

## Competing interests

The authors declare that they have no competing interests.

## Authors’ contribution

SM (study design, data collection, analysis and interpretation, manuscript writing). YH (study design, data collection, analysis and interpretation, manuscript edition). HK (study design, data collection and analysis). NT (study design, data collection and analysis). AS (study design, data collection and analysis). EO (study design, data analysis and interpretation). SM (study design, data analysis and interpretation). TH (study design, data analysis and interpretation). MM (study design, data analysis and interpretation). All authors read and approved the final manuscript.

## Pre-publication history

The pre-publication history for this paper can be accessed here:

http://www.biomedcentral.com/1471-2466/14/79/prepub

## Supplementary Material

Additional file 1: Figure S1 Acute cigarette smoke model **A**.**B**. Apoptosis in the lungs. ssDNA-positive or cleaved caspase-3-positive cells (arrow) in the alveolar septa are shown. **C**. Airway mucus overproduction. PAS positive cells were not detected in both strains. **Figure S2.** p38 MAPK activation. **A**. In a preliminary time course experiment (n = 1), the phosphorylation of p38 MAPK in the lungs was confirmed at 0.25, 1, 3, and 6 h after the start of CS exposure in C57BL/6 mice, but not in NZW mice. The phosphorylation of ERK and SAPK/JNK was noted in both strains. **B**. Chronic CS exposure did not affect phosphorylation of p38 MAPK in both strains (n = 3). p-p38 MAPK, phosphorylated-p38 MAPK; t-p38 MAPK, total p38 MAPK. C.D. Both acute **(C)** and chronic **(D)** CS exposure caused a marked increase in the number of phosphorylated p38-positive cells (arrow) in the alveolar walls of C57BL/6 mice, but not in NZW mice. **E**-**H**. Acute CS exposure did not affect lung mRNA expressions of p38 MAPK **(E)**, MAPKAPK-2 **(F)**, MKK3 **(G)**, and MKK6 (H) in both strains (n = 6). **I.** Chronic CS exposure up-regulated p38 MAPK mRNA in C57BL/6 mice but not in NZW mice (n = 6). *p < 0.05 compared with corresponding non-smoke groups. †*p* < 0.05 compared with C57BL/6 smoke groups. **Figure S3.** chronic cigarette smoke model. **A**.**B**. Apoptosis in the lungs. ssDNA-positive or cleaved caspase-3-positive cells (arrow) in the alveolar septa are shown. **C**. Airway mucus overproduction. PAS positive cells were not detected in both strains. **Figure S4.** acute cigarette smoke model (therapeutic model). There was no significant difference in the bronchoalveolar lavage cell differential (n = 6): macrophage **(A)**, neutrophil **(B)**, and lymphocyte **(C)**. Click here for file
