# Supplementary material for: p38 mitogen-activated protein kinase determines the susceptibility to cigarette smoke-induced emphysema in mice
Source: BMC Pulm Med. 2014 May 7;14:79. doi: 10.1186/1471-2466-14-79 (PMC4024315; doi:10.1186/1471-2466-14-79)

Supplementary Figure 1

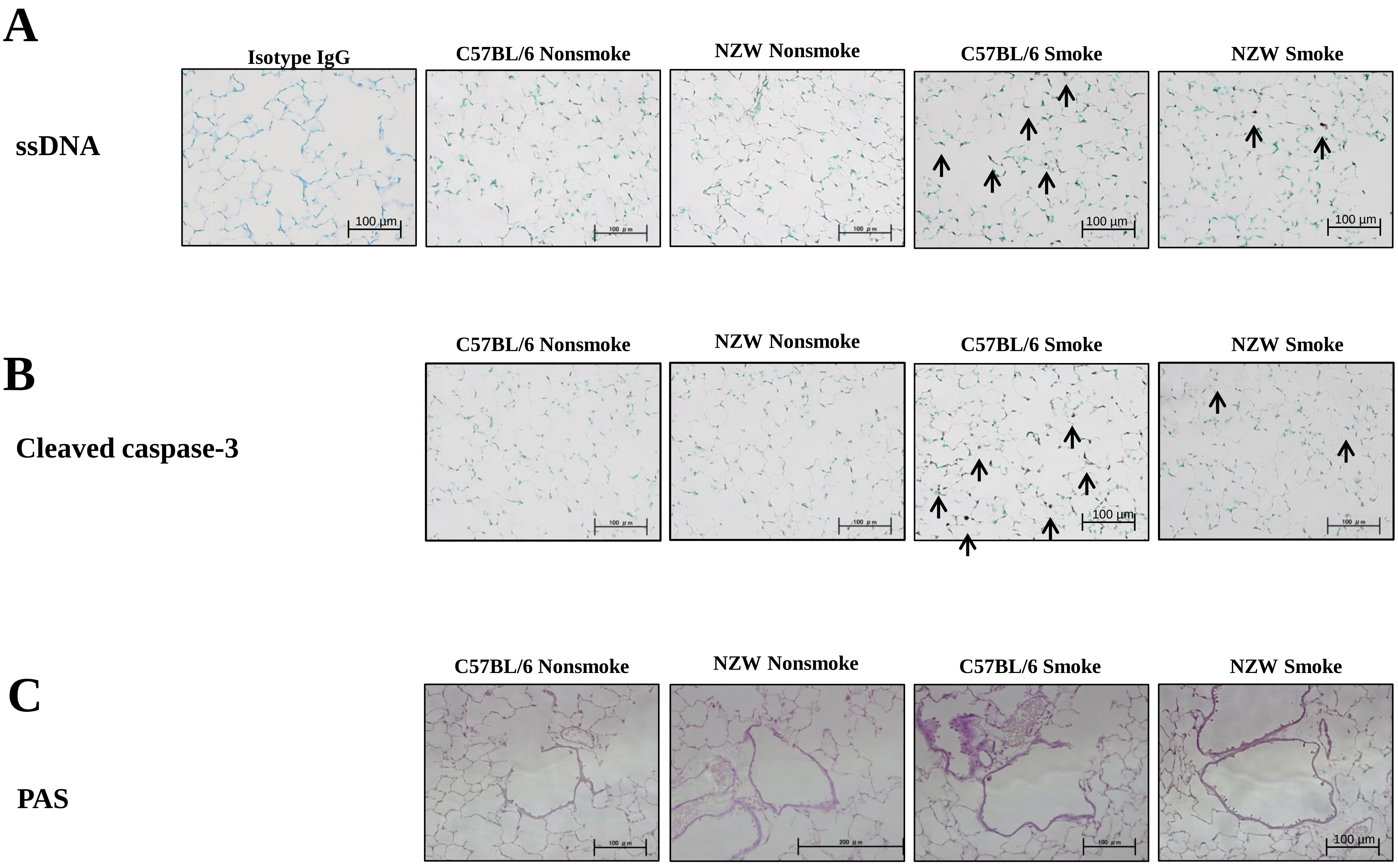

Supplementary Figure 2

A

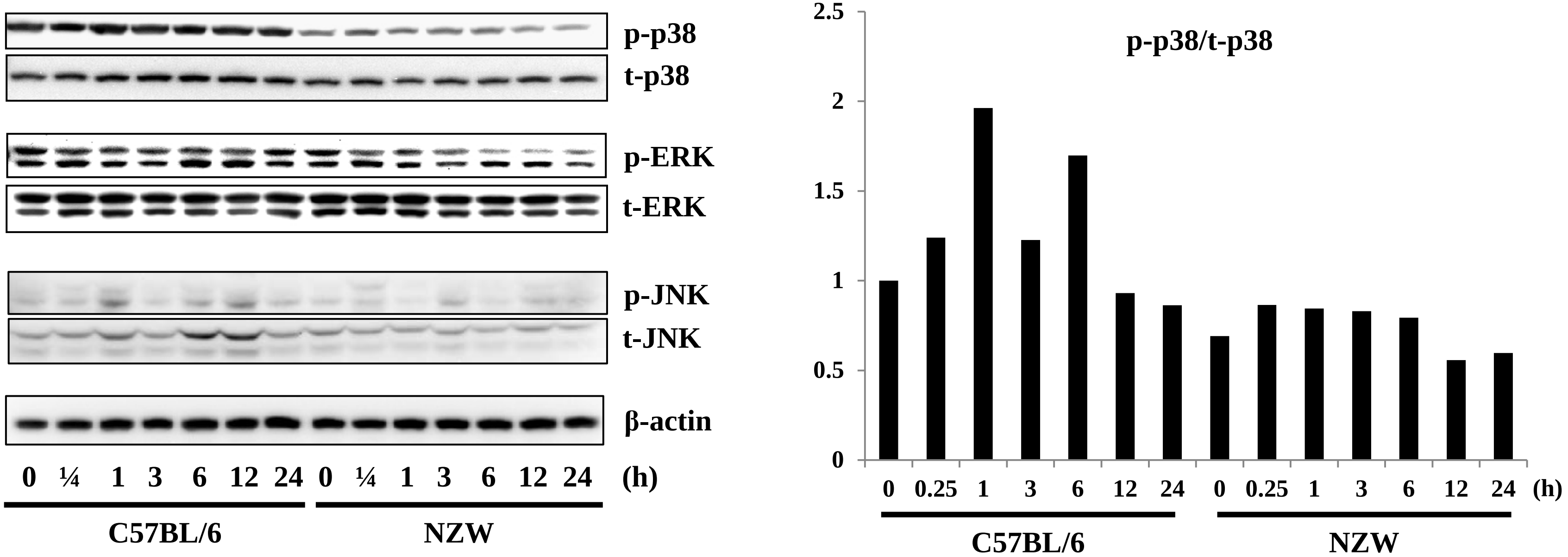

B

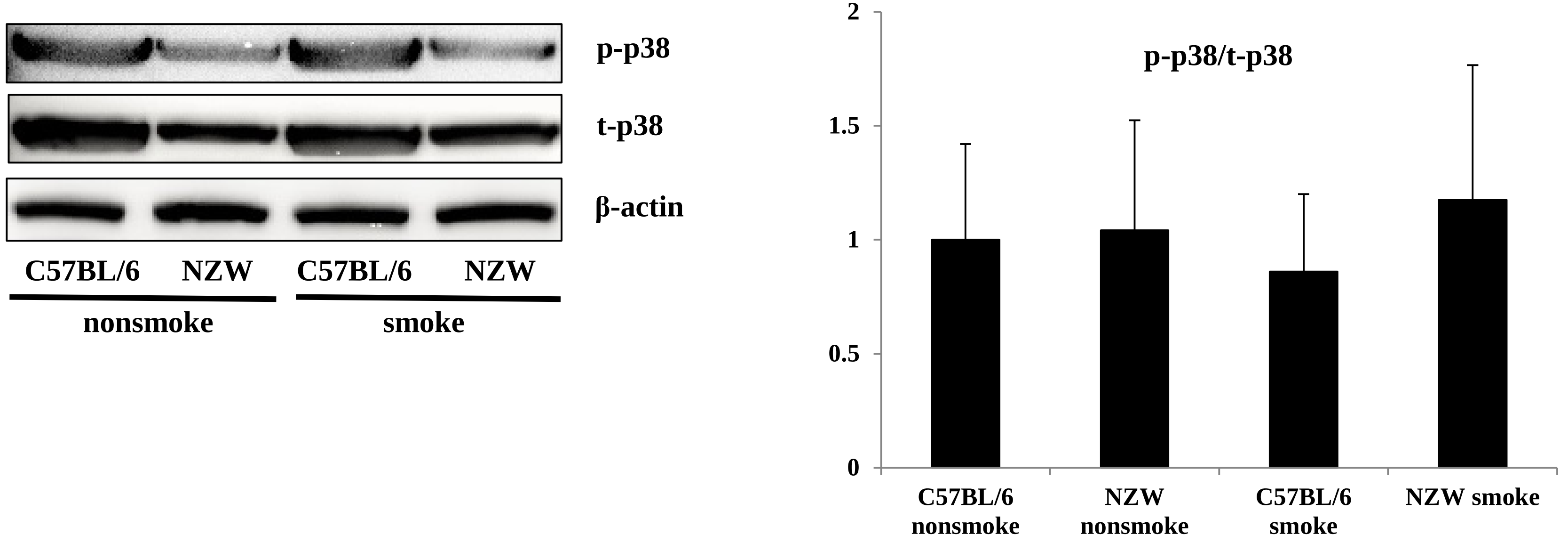

Supplementary Figure 2 (continued)

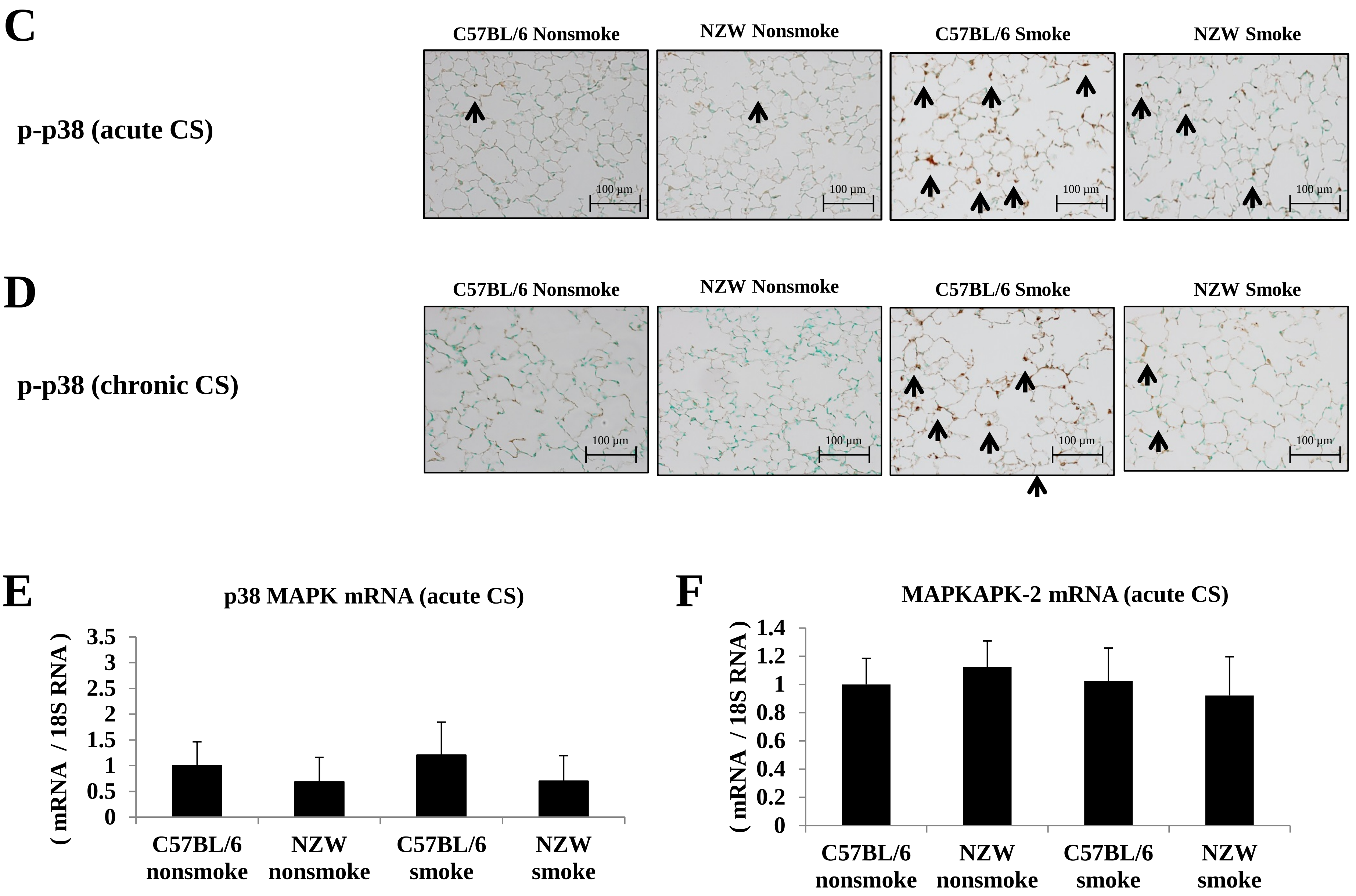

Supplementary Figure 2 (continued)

G

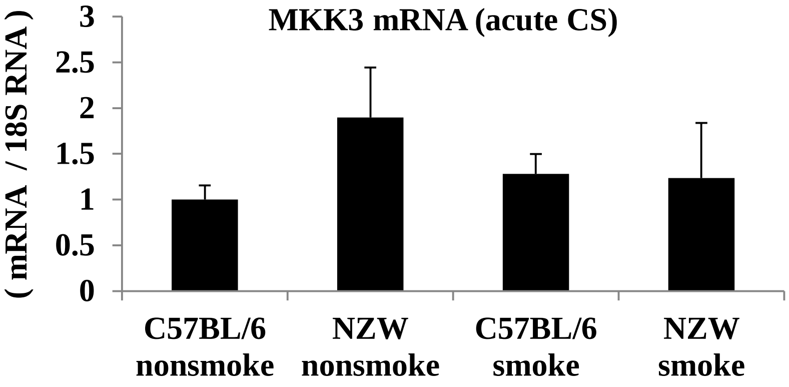

H

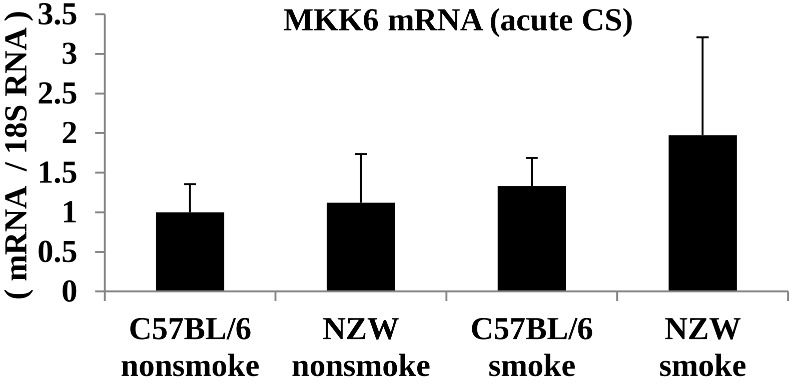

I

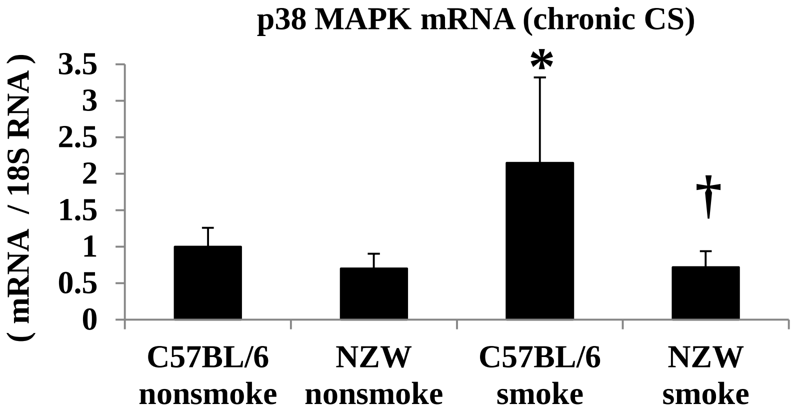

Supplementary Figure 3

A

ssDNA

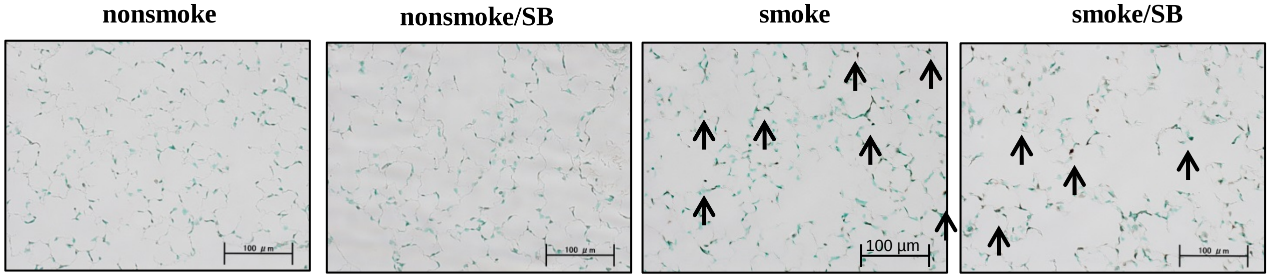

B

Cleaved caspase-3

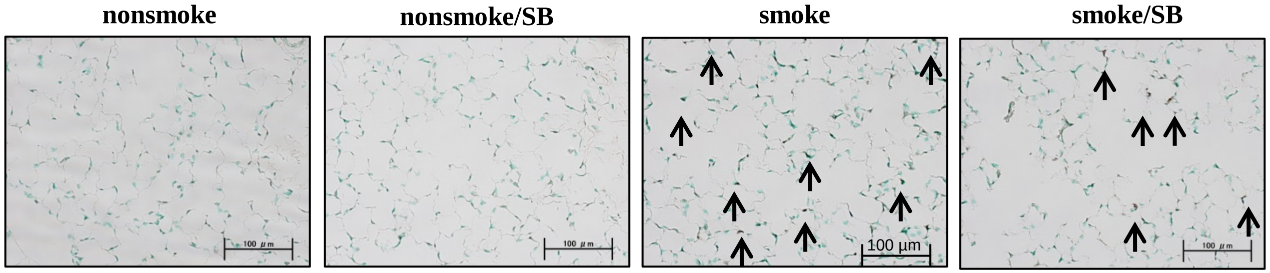

C

PAS

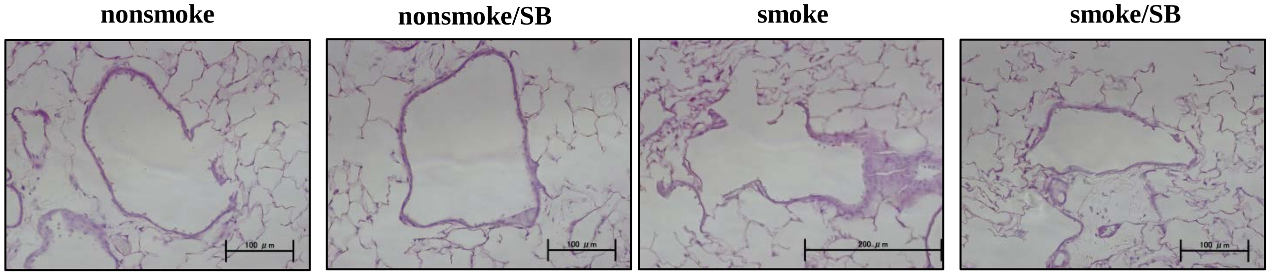

Supplementary Figure 4

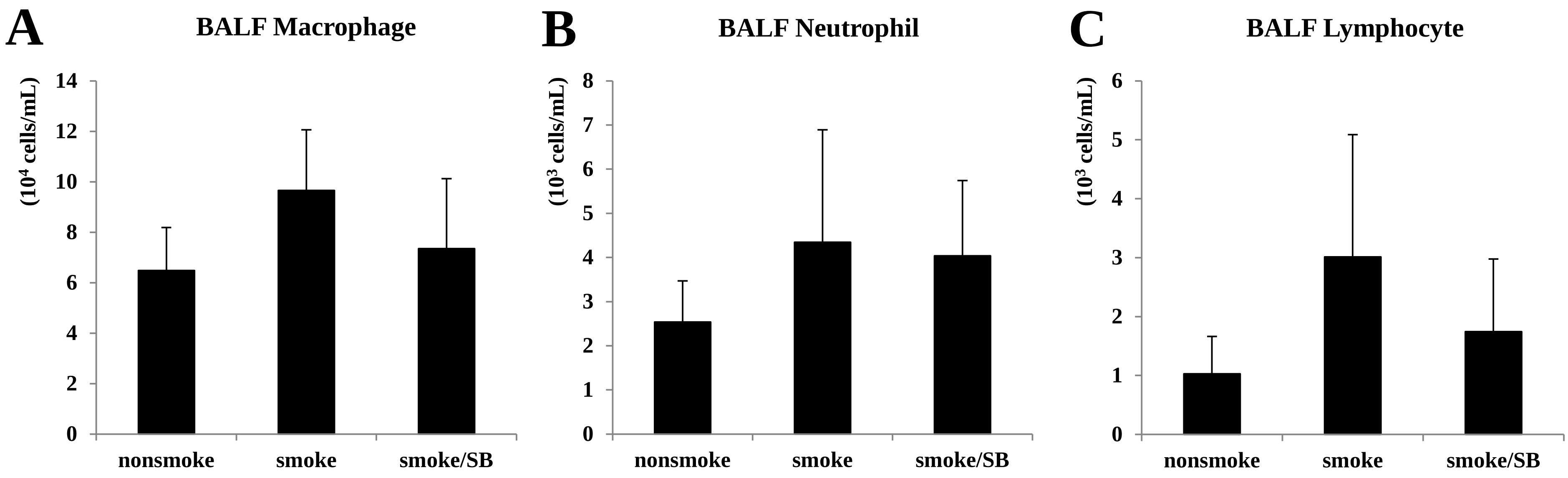

Supplement: Additional file 1: Figure S1 — Acute cigarette smoke model A.B. Apoptosis in the lungs. ssDNA-positive or cleaved caspase-3-positive cells (arrow) in the alveolar septa are shown. C. Airway mucus overproduction. PAS positive cells were not detected in both strains. Figure S2. p38 MAPK activation. A. In a preliminary time course experiment (n = 1), the phosphorylation of p38 MAPK in the lungs was confirmed at 0.25, 1, 3, and 6 h after the start of CS exposure in C57BL/6 mice, but not in NZW mice. The phosphorylation of ERK and SAPK/JNK was noted in both strains. B. Chronic CS exposure did not affect phosphorylation of p38 MAPK in both strains (n = 3). p-p38 MAPK, phosphorylated-p38 MAPK; t-p38 MAPK, total p38 MAPK. C.D. Both acute (C) and chronic (D) CS exposure caused a marked increase in the number of phosphorylated p38-positive cells (arrow) in the alveolar walls of C57BL/6 mice, but not in NZW mice. E-H. Acute CS exposure did not affect lung mRNA expressions of p38 MAPK (E), MAPKAPK-2 (F), MKK3 (G), and MKK6 (H) in both strains (n = 6). I. Chronic CS exposure up-regulated p38 MAPK mRNA in C57BL/6 mice but not in NZW mice (n = 6). *p < 0.05 compared with corresponding non-smoke groups. †p < 0.05 compared with C57BL/6 smoke groups. Figure S3. chronic cigarette smoke model. A.B. Apoptosis in the lungs. ssDNA-positive or cleaved caspase-3-positive cells (arrow) in the alveolar septa are shown. C. Airway mucus overproduction. PAS positive cells were not detected in both strains. Figure S4. acute cigarette smoke model (therapeutic model). There was no significant difference in the bronchoalveolar lavage cell differential (n = 6): macrophage (A), neutrophil (B), and lymphocyte (C). [file 1471-2466-14-79-S1.pdf]
